# Biased alternative polyadenylation in human tissues

**DOI:** 10.1186/gb-2005-6-12-r100

**Published:** 2005-11-28

**Authors:** Haibo Zhang, Ju Youn Lee, Bin Tian

**Affiliations:** 1 Department of Biochemistry and Molecular Biology, New Jersey Medical School, University of Medicine and Dentistry of New Jersey, 185 South Orange Avenue, Newark, NJ 07101-1709, USA

## Abstract

**Background:**

Alternative polyadenylation is one of the mechanisms in human cells that give rise to a variety of transcripts from a single gene. More than half of the human genes have multiple polyadenylation sites (poly(A) sites), leading to variable mRNA and protein products. Previous studies of individual genes have indicated that alternative polyadenylation could occur in a tissue-specific manner.

**Results:**

We set out to systematically investigate the occurrence and mechanism of alternative polyadenylation in different human tissues using bioinformatic approaches. Using expressed sequence tag (EST) data, we investigated 42 distinct tissue types. We found that several tissues tend to use poly(A) sites that are biased toward certain locations of a gene, such as sites located in introns or internal exons, and various sites in the exon located closest to the 3' end. We also identified several tissues, including eye, retina and placenta, that tend to use poly(A) sites not frequently used in other tissues. By exploring microarray expression data, we analyzed over 20 genes whose protein products are involved in the process or regulation of mRNA polyadenylation. Several brain tissues showed high concordance of gene expression of these genes with each other, but low concordance with other tissue types. By comparing genomic regions surrounding poly(A) sites preferentially used in brain tissues with those in other tissues, we identified several *cis*-regulatory elements that were significantly associated with brain-specific poly(A) sites.

**Conclusion:**

Our results indicate that there are systematic differences in poly(A) site usage among human tissues, and both *trans*-acting factors and *cis*-regulatory elements may be involved in regulating alternative polyadenylation in different tissues.

## Abstract 2

Bioinformatic analyses of the occurrence and mechanism of alternative polyadenylation in different human tissues reveals systematic differences among tissues and suggests the involvement of both *trans*- and *cis*-regulatory elements.

## Background

Polyadenylation is essential for the 3'-end formation of most mRNAs in eukaryotes. It involves two tightly coupled steps, cleavage of a nascent mRNA and polymerization of a poly(A) tail at the 3' end of the cleaved RNA. An array of factors are involved in the process, including factors that seem to be exclusively involved in polyadenylation, such as cleavage-polyadenylation specificity factor (CPSF), cleavage stimulatory factor (CstF), cleavage factors (CFs) I and II, and poly(A) polymerase (PAP), and factors that are involved in both polyadenylation and other cellular processes, including transcription and mRNA splicing, such as RNA polymerase II, Symplekin [1,2], PC4 [3], Ssu72 [4], heterogeneous nuclear ribonucleoprotein (hnRNP) F [5], hnRNP H/H' [6], U2AF65 [7], U1A [8-10], polypyrimidine tract binding protein (PTB) [11], and SRp20 [12]. The fact that some factors are involved in both polyadenylation and transcription and mRNA splicing supports the notion that these processes are tightly coupled [13,14]. In addition, the processing efficiency of polyadenylation has a direct impact on the amount of mRNAs produced [15]. Abnormal processing efficiency can lead to human diseases such as thrombophilia [16].

Both biochemical and bioinformatic methods have been applied to the identification of *cis*-regulatory elements (or *cis *elements) for polyadenylation. The polyadenylation signal (PAS) is located 10 to 35 nucleotides (nt) upstream of the cleavage site, and serves as the binding site for CPSF. It is usually AAUAAA or a single nucleotide variant [17,18]. U/GU-rich elements are located within approximately 40 nt downstream of the cleavage site [19,20], serving as the binding site for CstF. In addition, several auxiliary upstream elements and downstream elements have been found in viral or cellular genes that can promote or repress polyadenylation [21-24].

Recent studies have shown that over half of the human genes have multiple polyadenylation sites (poly(A) sites) [18,25]. Like alternative initiation and alternative splicing, alternative polyadenylation (Alt-PA) contributes to the complexity of the transcriptome in human cells by producing mRNAs with different 3' untranslated regions (3'UTRs) and/or encoding variable protein isoforms [15]. The regulation of 3'UTRs by Alt-PA can have a different impact on the mRNA metabolism, as 3'UTRs can contain various regulatory elements, such as AU-rich elements responsible for mRNA stability [26,27] and miRNA target sequences involved in the regulation of mRNA translation [28-30]. The effect of Alt-PA on protein coding is usually coupled with alternative splicing [15], and has been demonstrated for several genes. Well-studied examples include regulation of the IgM heavy chain gene [31] and regulation of calcitonin/calcitonin gene-related peptide [32,33]. Many poly(A) sites are preferentially used in certain tissues and under specific cellular conditions [15,34]. It is not known, however, whether the pattern of poly(A) site usage is systematically different among human tissues, which could result in coordinate regulation of 3'UTRs or encoded proteins for a large number of genes.

Here we describe our effort to study tissue-specific Alt-PA events using bioinformatic approaches. Using expressed sequence tag (EST) data and a newly developed method named GAUGE (for global study of poly(A) site usage by gene-based EST vote), we investigated 42 tissue types. We found that several tissues tend to use poly(A) sites that are biased toward certain locations of a gene, that is, 5' or 3' poly(A) sites. For poly(A) sites located in the 3'-most exon, biased usage was found in the nervous system, brain, pancreatic islet, ear, bone marrow, uterus, retina, placenta, ovary, and blood. For poly(A) sites located in introns or internal exons, biased usage was observed in cerebrum, soft tissue, pancreas, lung, prostate, skin, placenta, esophagus, eye, retina, and blood. In addition, we found that eye, retina, and placenta tend to use poly(A) sites not preferred in other tissues. Using microarray expression data of polyadenylation-related protein factors, we found that several brain tissues have high concordance with each other, and low concordance with other tissues. Finally, we identified several *cis *elements that are preferentially associated with brain-specific poly(A) sites. Taken together, our data suggest that systematic bias of Alt-PA occurs in several human tissues, and both *cis *elements and *trans*-acting factors are responsible for regulating Alt-PA.

## Results

### Positional preference of polyadenylation in human tissues

**Figure 1:**
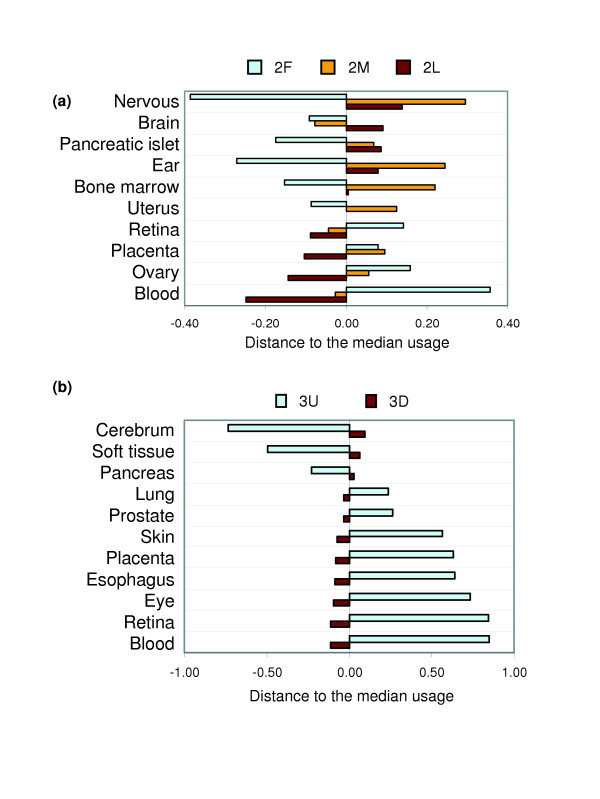
Tissue-specific positional preference of poly(A) site usage. **(a) **Tissue-specific positional preference of type II genes. **(b) **Tissue-specific positional preference of type III genes. Distance to the median was calculated as (observed usage - median usage)/median usage. Only tissues with significant *p* values (<0.05, Chi-squared test) are shown here. 2F, 2M, and 2L are the poly(A) sites closest to the 5' end, middle, and closest to the 3' end in a type II gene, respectively. 3U and 3D are poly(A) sites located upstream of the exon closest to the 3' end and poly(A) sites in the exon closest to the 3' end in a type III gene, respectively.

We have previously shown that approximately 54% of human genes have multiple poly(A) sites [18]. Poly(A) sites can be located in various regions of a gene, including introns, internal exons, and 3'-most exons [18,25]. To address whether there are positional preferences of Alt-PA in human tissues, we evaluated tissue-specific poly(A) site usage based on the relative position of a poly(A) site in a gene. We have previously classified human genes into three types according to the locations of their poly(A) sites [18] (also shown in Figure 1 in Additional data file 1). Briefly, genes with only one poly(A) site are classified as type I genes, genes with multiple poly(A) sites all in the 3'-most exon as type II genes, and genes with poly(A) sites located in introns or internal exons as type III genes. Alt-PA of type II genes may result in mRNAs with variable 3'-UTRs, and the usage of poly(A) sites located in introns or internal exons of type III genes can potentially have an impact on protein sequence or lead to mRNAs with no in-frame stop codons. Thus, by investigating the poly(A) site usage of type II and III genes, one can address the question of whether Alt-PA leads to variable 3'-UTRs or protein products in certain tissue types. To this end, we classified poly(A) sites of type II genes into 2F (the 5'-most poly(A) site), 2L (the 3'-most poly(A) site), and 2M (middle poly(A) sites between 2F and 2L); and classified poly(A) sites of type III genes into 3U (poly(A) sites located upstream of the 3'-most exon) and 3D (poly(A) sites located in the 3'-most exon) (Figure 1 in Additional data file 1).

Based on the EST tissue information obtained from the UniGene database [35] and assisted by the cDNA library classification made by Yeo *et al*. [36], we first grouped cDNA libraries into tissue types. In order to make quantitative comparisons, we used only non-normalized cDNA libraries. In total, we grouped 609 non-normalized cDNA libraries into 42 tissue types, corresponding to 86,495 poly(A/T)-tailed ESTs (Table 1 in Additional data file 1). To examine tissue-specific usage of the three types of poly(A) sites in type II genes (2F, 2M, and 2L) and the two types of poly(A) sites in type III genes (3U and 3D), we developed a method named GAUGE as follows. Type II or type III genes cast votes for the usage of different poly(A) site types in every tissue. The votes were based on the number of supporting ESTs for certain poly(A) sites, for example, 2F, 2M, or 2L. To account for the difference of expression levels among genes, the number of ESTs for each poly(A) site type was divided by the total number of ESTs supporting each gene. Thus, for each gene, the sum of the vote for all site types equals 1. The percentage of usage of a poly(A) site type in a tissue can then be measured by the votes cast by all genes expressed in that tissue divided by the number of genes. We then carried out Chi-squared tests to measure the bias of usage of different types of sites in each tissue. For each poly(A) site type in a tissue, its percent of usage was compared with the median percent of usage of all tissues. The difference was normalized to the median and called distance (Figure 1). Complete lists of values for all tissues are provided in Tables 2 and 3 in Additional data file 1.

Several tissues were found to have significantly biased (*p* value < 0.05) usage of certain poly(A) sites of type II and/or type III genes (Figure 1). Increased usage of 5'-most poly(A) sites (2F) was observed for placenta, retina, blood, and ovary. These tissues were also found to have decreased usage of 3'-most poly(A) sites (2L), suggesting a shift of usage from 3' poly(A) sites to 5' poly(A) sites. In bone marrow, uterus, ear, brain, the nervous system, and pancreatic islet, however, the preference is the opposite, with a decreased usage of 5'-most poly(A) sites (2F) and increased usage of the 3' poly(A) sites (2M and 2L), suggesting a shift of usage from 5' poly(A) sites to 3' poly(A) sites. Similarly, placenta, eye, prostate, skin, esophagus, retina, blood, and lung were found to have significantly increased usage of poly(A) sites located upstream of the 3'-most exon (3U), whereas cerebrum, soft tissue, and pancreas were found to have the opposite, biased to 3D, preference in poly(A) site usage. Interestingly, placenta, retina, and blood were found to have positional preference of poly(A) sites for both type II and type III genes, and the preferences were both toward the 5' poly(A) sites (2F and 3U).

### Tissues with distinct poly(A) site usage

We further asked the question whether some tissues tend to use poly(A) sites that are not frequently used in other tissues. The overall usage of a poly(A) site could be considered as its 'strength' [37]. Accordingly, frequently used poly(A) sites were called 'strong' sites, whereas less frequently used sites were called 'weak' sites. Presumably, strong sites are associated with favorable *cis *elements for polyadenylation, and weak sites either lack these elements or are associated with repressing elements. Our goal was to identify tissues that had significantly biased usage of strong or weak poly(A) sites. To this end, we classified 22,865 poly(A) sites from 7,524 alternatively polyadenylated human genes in our polyA_DB database [38] into strong and weak sites by their supporting ESTs from non-normalized cDNA libraries. In order to have robust results, we used three cutoffs for the classification, 60%, 75%, and 90%. For each gene at a given cutoff, the poly(A) site with the percent of supporting ESTs above the cutoff was classified as a strong site. If there was a strong poly(A) site, other sites of the same gene were classified as weak sites. It is noteworthy that we used ESTs derived from a large number of cDNA libraries, corresponding to 42 tissue types (Table 1 in Additional data file 1). Thus, the classification should not be biased by ESTs from certain tissue types, and the strength should reflect poly(A) site usage in most tissues, that is, strong sites are 'globally preferred', whereas weak sites are not.

**Figure 2:**
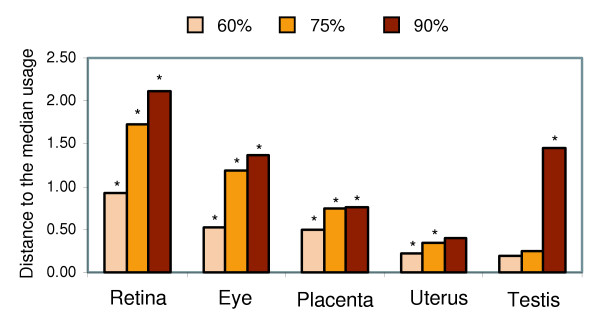
Tissue-specific strong and weak poly(A) site usage. For each tissue, three bars represent the distance to the median usage according to three different cutoffs for the classification of strong and weak sites, for example, 60%, 75% and 90%. For each gene at a given cutoff, the poly(A) site with the percent of supporting ESTs above the cutoff was classified as a strong site. If there was a strong poly(A) site, other sites of the same gene were classified as weak sites. Only values for the weak sites are shown. Significant ones (*p* value < 0.05, Chi-squared test) are marked with asterisks.

To examine the usage of strong and weak poly(A) sites, we applied the GAUGE method described above with the modification that genes voted for strong and weak sites. Among 42 tissues investigated, retina, eye, placenta, uterus, and testis had significant biases (*p* value < 0.05) using at least one of the cutoffs (Figure 2; complete lists of values for all tissues using different cutoffs are provided in Tables 4-6 in Additional data file 1). All the significant biases were toward the weak sites. Among these, retina, eye, and placenta had consistent biases at all cutoffs. Interestingly, retina and placenta were found to be biased to 2F poly(A) sites and eye, retina, and placenta were found to be biased to 3U poly(A) sites (see above), indicating that eye, retina, and placenta use distinct sets of poly(A) sites that are of the types 2F and 3U. As testis was found to be biased to weak sites only using the 90% cutoff, it appears that a set of genes expressed in the testis select poly(A) sites that are strongly preferred, if not uniquely used, in testis. Taken together, our observations of positional bias and distinct poly(A) site usage suggest that there is biased usage of poly(A) sites in certain human tissues. As these tissue-specific preferences were observed on a global rather than gene-specific level, the mechanism for these biases may lie in tissue-specific regulation of expression of certain polyadenylation factors.

### Differential expression of polyadenylation-related protein factors among tissues

We then wished to address whether there were differences in gene expression of *trans*-acting factors among different tissues, which might be responsible for the observed tissue-specific poly(A) site usage. To this end, we obtained mRNA expression data from two microarray studies [39,40], involving U95Av2 and U133A Affymetrix GeneChips (Affymetrix, Santa Clara, CA, USA), respectively. To cross-validate the data, we focused on 25 tissue types whose gene expression was analyzed in both studies. From the literature we identified 26 factors that were known or had been suggested to play roles in nuclear polyadenylation (Table 1). Of these 26 factors, 21 had probes on both U95Av2 and U133A microarrays. In addition, U133A had probes for two additional genes, and three factors had no probes on either microarray.

**Figure 3:**
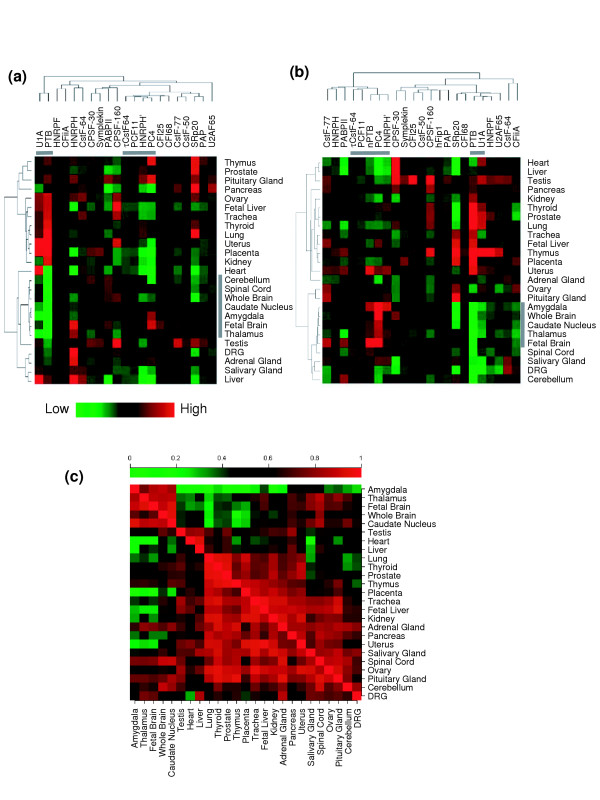
Gene expression of polyadenylation factors. **(a) **Two-way hierarchical clustering of the U95Av2 data using 21 polyadenylation factors. **(b) **Two-way hierarchical clustering of the U133A data using 23 polyadenylation factors. See Table 1 for polyadenylation factors in each dataset. Tissues and genes that are consistently clustered together in both datasets are marked by gray lines. **(c) **Correlation of mRNA expression levels of 21 polyadenylation-related factors across 25 human tissues (upper diagonal, data from U133A; lower diagonal, data from U95Av2). Based on the scale displayed on top of the figure, small squares are colored to represent the extent of correlation between mRNA expression levels of the 21 genes in each pair of human tissues. DRG, dorsal root ganglion.

Microarray data were first normalized to the 75 percentile within each tissue, and were then subjected to hierarchical cluster analysis based on the Pearson correlations of expression levels of polyadenylation factors. As shown in Figure 3, there are conspicuous differences among different tissues. Interestingly, several brain tissues, including amygdala, thalamus, fetal brain, whole brain, and caudate nucleus, form a distinct cluster in both datasets (Figure 3a,b). To assess the robustness of cluster analysis, we compared the results of the U95Av2 and U133A studies. We calculated the Pearson correlation coefficients (*r*) between all pairs of 25 tissues using the expression of the 21 polyadenylation factors. The *r *values ranged from -1 to 1, with values close to 1 indicating high concordance, and values close to -1 indicating low concordance. We then clustered tissues based on *r *values obtained from both U95Av2 and U133A datasets, and presented the values in a heatmap (Figure 3c). We found that the expression values of the 21 polyadenylation factors were well correlated (*r *> 0.75) for most tissues, suggesting consistency of these two studies with respect to the factors investigated. As expected, a distinct cluster containing several brain tissues (amygdala, thalamus, caudate nucleus, fetal brain, and whole brain) can be discerned (average *r*-value 0.87 within the cluster), which showed low concordance with other tissues (average *r*-value 0.55 between the cluster and other tissues). The clustering result to some extent agrees with our studies using ESTs. For example, lung, ovary, placenta, and prostate, which were among the 25 tissues in the microarray studies, had significant positional bias towards 5' poly(A) sites (2F or 3U; Figure 1), and brain and cerebrum had a statistically significant positional preference for 3' poly(A) sites (2L or 3D; Figure 1). Consistent with these observations, expression data from brain tissues correlated poorly (mean *r*-value of 0.41) with those from placenta, lung, ovary, or prostate (Figure 3c).

**Figure 4:**
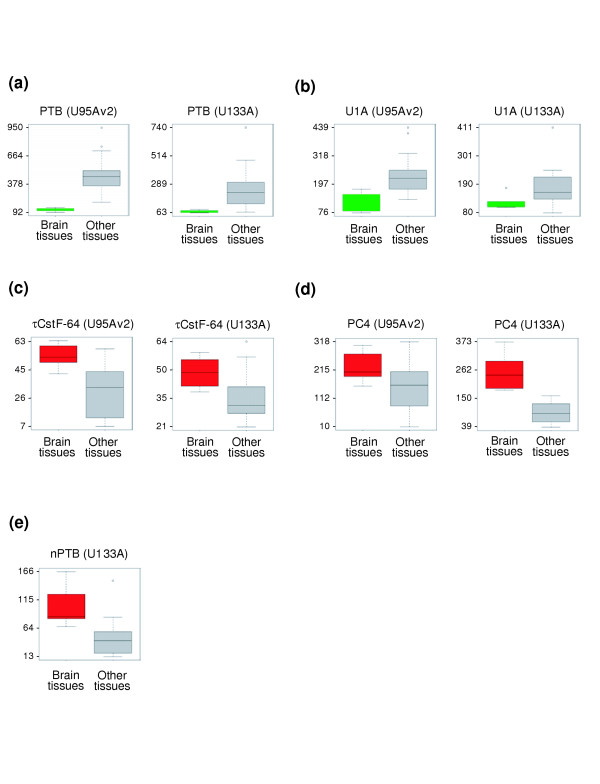
Boxplots of mRNA expression of several factors in brain and other tissues. **(a) **PTB, **(b) **U1A, **(c) **τCstF-64, **(d) **PC4, and **(e) **nPTB. All factors except nPTB were present in both the U95Av2 and U133A datasets. Brain tissues include amygdala, thalamus, caudate nucleus, fetal brain, and whole brain. Expression values lower in brain tissues than other tissues are in green; and those higher in brain tissues are in red.

Two studies showed that, of the 21 genes, U1A and PTB had a similar expression pattern across tissues, as did PC4, PCF11, τCstF-64, and hnRNP H'. The difference between brain tissues and others was mainly attributable to the expression of four genes: The expression of PTB and U1A was consistently lower in brain tissues than in other tissues (*p* value < 0.05 by *t* test; Figure 4a,b), whereas the relative expression levels of PC4 and τCstF-64 were consistently higher in brain tissues (*p* value < 0.05 by *t* test; Figure 4c,d). Comparisons of expression levels between brain tissues and other tissues for the rest of the 17 factors, however, did not show such differences in either U95Av2 or U133A datasets (Figure 2 in Additional data file 1). In addition, neural polypyrimidine tract binding protein (nPTB), whose expression value was only available in the U133A study, was also found to be preferentially expressed in brain tissues (Figure 4e), consistent with previous reports that it was primarily expressed in neuronal cells.

### *Cis *elements associated with poly(A) sites preferentially used in brain tissues

**Figure 5:**
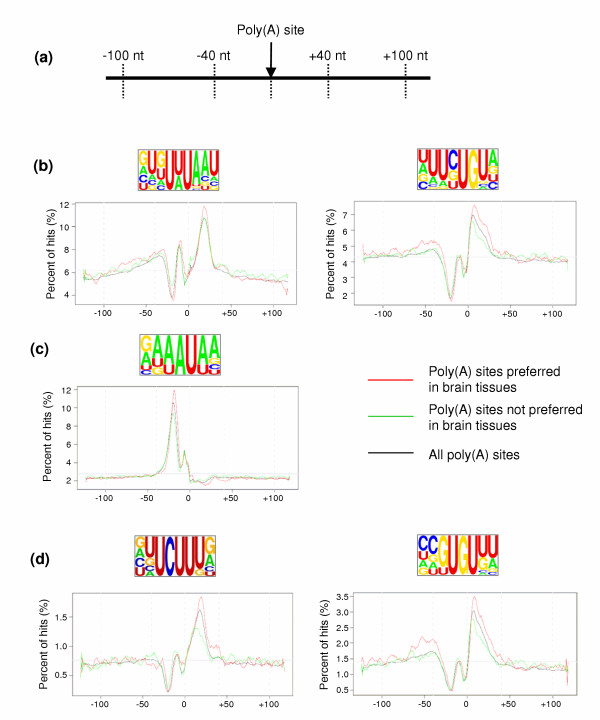
Brain-specific over-represented *cis *elements. **(a) **Schematic of the four poly(A) regions investigated. **(b) **Identified *cis *elements in -100/-41. **(c) **Identified *cis *elements in -40/-1. **(d) **Identified *cis *elements in +1/+40. Logos are shown for *cis *elements. Under each logo is the percent of hits for the corresponding *cis *element in poly(A) sites preferred in brain tissues (red), poly(A) sites not preferred in brain tissues (green), and all poly(A) sites (black). In the graphs, the y-axis is the percent of hits, and the x-axis is the distance to the poly(A) site. Horizontal dotted lines are the average value, and vertical dotted lines are the -100, -40, +40, +100 nt positions.

The well-established paradigm in gene regulation is that *trans*-acting factors and *cis *elements work in concert. Our observations of biased Alt-PA in certain human tissues prompted us to investigate candidate *cis *elements. We focused on brain tissues in this study, as they were found to have biased usage of poly(A) sites, and several brain tissues had similar gene expression patterns for a number of polyadenylation factors, which could make it straightforward to correlate the expression of *trans *factors and the usage of identified *cis *elements. To select *cis *elements that are preferentially present near poly(A) sites used in brain tissues, we first selected poly(A) sites belonging to genes that have multiple poly(A) sites and are expressed in brain tissues. We then focused on the -100/+100 nt genomic region of poly(A) sites (the poly(A) site is arbitrarily set at position 0) for identification of *cis *elements. This is based on previous studies that indicated the -100/+100 nt region had a different nucleotide composition to regions further upstream (<-100) or downstream (>+100) of a poly(A) site [18,37]. We divided the genomic sequence surrounding a poly(A) site into four regions: -40/-1 nt, where the PAS is located; +1/+40 nt, where U/GU-rich elements are usually located; -100/-41 nt, where auxiliary upstream elements may be located; and +41/+100 nt, where auxiliary downstream elements may be located (Figure 5a). In addition, we used the -300/-200 and +200/+300 regions as control regions, which, based on our current knowledge of *cis *elements for polyadenylation, should contain very few, if any, regulatory elements for polyadenylation. To identify *cis *elements in brain-specific poly(A) sites, we took an approach that is similar to the method described in [24]. Briefly, hexamers (4,096 in total) were assigned with two scores: *z*_*un*_, the difference between the frequency of occurrence in a specific sequence region, for example, -100/-41, of poly(A) sites used in brain tissues (a total of 2,495 sites) and those not used in the brain (a total of 3,297 sites); and *z*_*pc*_, the difference between the frequency of occurrence in a specific poly(A) region and the frequency of occurrence in control regions. We then selected hexamers with both *z*_*un *_and *z*_*pc *_greater than 2.5. A z score of 2.5 corresponds to a *p* value of approximately 0.01 in a normal distribution. To avoid the identification of *cis *elements that are globally preferred, we filtered out hexamers with *z*_*sw *_> 2.5, where *z*_*sw *_is the difference between the frequency of occurrence in a specific sequence region of strong poly(A) sites and that of weak poly(A) sites, using 75% as the cutoff for classification of strong and weak sites. Selected hexamers were grouped by their mutual similarities, and groups with more than three hexamers were used to build sequence logos. In addition, position-specific scoring matrices (PSSMs) were derived from the logos, and used to search corresponding *cis *elements in poly(A) regions.

We identified five putative elements that were significantly over-represented in various regions of poly(A) sites preferentially used in brain tissues (Figure 5). Among these, a GU element (Figure 5d, right panel) was identified in region +1/+40, which seems to be the binding site for CstF-64. GU elements should be general enhancers for polyadenylation. As we filtered out hexamers that are significantly associated with strong poly(A) sites, the fact that a GU element still remains indicates that the GU element is strongly biased to poly(A) sites used in brain tissues. This notion is in line with the difference between the percent of hits profile of the GU element in brain specific poly(A) sites compared to non-brain poly(A) sites (Figure 5d, right panel). As the expression of CstF-64 is similar between brain tissues and other tissues and the expression of τCstF-64 is significantly higher in brain tissues, the identified element could be the preferred binding site of τCstF-64. This prediction, however, needs to be validated in wet lab experiments. In addition, we found that the UCUUU element (Figure 5d, left panel) was over-represented in region +1/+40. UCUUU is known to be the binding site of PTB [11,41]. Interestingly, the UUC/GUG element identified in the -100/-41 region (Figure 5b, right panel) also resembles PTB binding sites. As shown by the microarray data, PTB expression is low in brain tissues, whereas the nPTB level is high. Thus, it will be interesting to examine whether nPTB binds to these *cis *elements and plays a role in poly(A) site selection in brain tissues. Furthermore, two other elements (Figure 5b, left panel and Figure 5c) seem to be related to U-rich elements and the AAUAAA PAS. Their significance is not clear, despite the fact that their percent of hits profiles differ between poly(A) sites preferred in brain tissues and those not preferred (Figure 5b,c). They could well be general regulatory elements that were not filtered out using *z*_*sw *_scores (see above). In line with this notion, both elements only had four supporting hexamers, whereas the GU element and UCUU element had five supporting hexamers, and the UUC/GUG element had seven supporting hexamers (Figure 3 in Additional data file 1).

## Discussion

We have detected biased poly(A) site usage in several human tissues using GAUGE. GAUGE was designed to detect systematic bias of poly(A) site usage in different tissues. The idea is that individual genes may not have statistical power for detection of overall trend, whereas significant patterns could emerge using a large number of genes. Although the numbers of cDNA libraries and ESTs for some tissue types were sufficient to allow us to make statistical conclusions, some others did not have enough numbers for sensitive detections, such as heart and thymus (Table 1 in Additional data file 1). If more ESTs become available, this approach could be carried out for these tissues in the future. On a similar note, an inherent limitation of our approach is that we could not assess the bias for individual genes due to lack of statistical power, which, at the current stage, is best addressed by wet lab experiments.

For poly(A) sites located in the 3'-most exon, the nervous system, brain, pancreatic islet, ear, bone marrow, and uterus tend to use 3' poly(A) sites, whereas retina, placenta, ovary, and blood tend to use 5' poly(A) sites. This observation indicates that genes may express mRNAs with longer 3'UTRs in certain tissues than in others, and the pattern is systematically controlled. Consistent with our observation, it has been suggested that brain tissues tend to express larger genes than other tissues [42], presumably due to the low mitotic activity of highly differentiated cells in the brain allowing more time to express long transcripts. Our data also suggest that each tissue type may have a defined 'program' to produce mRNAs with certain length. Given that 3'UTRs contain various RNA regulatory elements, it is conceivable that this mode of gene regulation could coordinately influence mRNA metabolism for a large number of genes. However, the exact impact of this systematic control needs to be explored in wet lab settings. In addition, lung, prostate, skin, placenta, esophagus, eye, retina, and blood were found to have higher usage of poly(A) sites located upstream of the 3'-most exon than other tissues. The usage of these poly(A) sites could result in truncated mRNAs without in-frame stop codons, or mRNAs encoding distinct protein isoforms. The coordinated regulation of poly(A) site usage could, therefore, lead to a switch in the expression of protein isoforms. As poly(A) sites located upstream of the 3'-most exon are next to introns and internal exons, regulation of this type of poly(A) sites is complicated by other factors, such as transcription and mRNA splicing. For example, both the IgM heavy chain gene [31] and the calcitonin/calcitonin gene-related peptide [32,33] gene switch protein products by using different poly(A) sites under certain cellular conditions. In both cases, alternative splicing was also shown to be involved.

We found that the expression of U1A, PC4, τCstF-64, PTB, and nPTB were significantly different between brain tissues and other tissues. The differences may contribute to the distinct Alt-PA pattern in the brain. It has been shown that brain tissues exhibit high levels of alternative splicing, especially exon skippings [36], which is consistent with our observation of a low expression level of PTB, a repressor of mRNA splicing [43], in brain tissues. It has also been shown that PTB can modulate polyadenylation efficiency by competing with CstF-64 for binding to downstream U/GU-rich elements [11]. nPTB shares high sequence homology with PTB [44,45] (Figure 4a in Additional data file 1), but its activity in regulating polyadenylation has not been studied. U1A can modulate polyadenylation by interacting with the poly(A) polymerase [10]. Furthermore, PC4 can regulate polyadenylation by interacting with CstF-64 [3]. τCstF-64 appears to be a paralog of CstF-64 (75% identity in protein sequence), which has been previously reported to be highly expressed in the brain and testis [46]. CstF-64 and τCstF-64 are highly homologous (>95% identity; Figure 4b in Additional data file 1) in both the amino-terminal RNA binding domain, which is responsible for interacting with U/GU-rich elements, and the carboxy-terminal 63 amino acid region, which has been implicated in binding to PC4 [3], indicating that the functions of CstF-64 and τCstF-64 may overlap extensively. Thus, nPTB and τCstF-64 appear to be functional homologs of PTB and CstF-64, respectively. Our observations that both nPTB and τCstF-64 mRNA levels are higher in brain tissues than other tissues, whereas the PTB mRNA level is lower in brain tissues and there is no difference in CstF-64 mRNA expression between brain tissues and other tissues (Figure 2 in Additional data file 1), indicate that brain tissues use a different set of genes to regulate splicing and polyadenylation, albeit their functions may be similar to their counterparts in other tissues.

In this study, we used brain tissues as a model to correlate the presence of *cis *elements and expression of *trans *factors. The reason to choose brain tissues is that biased usage of poly(A) sites was observed in brain tissues and high concordance of gene expression of polyadenylation factors was detected among several brain tissues. The latter is important as microarray data often contain noise. Using two datasets and several brain tissues gave us assurance as to the quality of the data. On the other hand, we only focused on known polyadenylation factors. Other protein factors that may also be involved in the regulation of polyadenylation were not examined in this study. Nevertheless, the significant presence of PTB and CstF-64 binding sites near poly(A) sites preferentially used in brain tissues correlates with high expression levels of nPTB and τCstF-64, which suggests a model where nPTB and τCstF-64 function cooperatively in poly(A) site selection in brain tissues. However, the exact details of the interactions need to be investigated in the future. In addition, the role of PC4 in regulating poly(A) site selection in the brain is to be examined, as a higher level of PC4 was observed in brain tissues versus other tissues. Furthermore, the same approach for identifying tissue-specific usage of *cis *elements can be applied to other tissue types. Ear, retina, and placenta will be particularly interesting to study, as they were found to use poly(A) sites that are not frequently used in other tissues and all three tissues tend to use 5' poly(A) sites.

## Materials and methods

### Datasets and resources

Genes with alternative poly(A) sites, their annotations including poly(A) positions and supporting EST evidence were obtained from polyA_DB [38]. General annotations of cDNA libraries were downloaded from the UniGene database [35]. A PERL script was used to determine whether a cDNA library is normalized or non-normalized. A cDNA library is classified as non-normalized if its annotation contains 'non-normaliz' or 'not normaliz', or does not contain the string 'normaliz' in any part of the annotation. Assisted by tissue annotations for cDNA libraries made by Yeo *et al*. [36], we grouped 609 non-normalized cDNA libraries into 42 tissue types. Microarray datasets [39,40] were downloaded from NCBI GEO [47]. Mappings of probe-sets to LocusLink IDs were obtained from the Affymetrix website [48].

### Identification of biased usage of poly(A) sites in human tissues by GAUGE

For genes with more than one poly(A) site, we used the number of supporting ESTs to classify strong or weak sites. To make robust assessment, we used three cutoffs for the classification, specifically, 60%, 75%, and 90%. For each cutoff, the poly(A) site with the percent of supporting ESTs above the cutoff was classified as a strong site. If there was a strong poly(A) site, other sites of the same gene were classified as weak sites. In addition, we required that the sum of ESTs for all weak sites must be above 1. Type II and type III genes were classified as previously described [18]. Poly(A) sites in type II genes were classified into 2F (the 5'-most poly(A) site), 2L (the 3'-most poly(A) site), and 2M (middle poly(A) sites between 2F and 2L); and poly(A) sites in type III genes were classified into 3U (poly(A) sites located upstream of the 3'-most exon) and 3D (poly(A) sites located in the 3'-most exon).

To study the usage of poly(A) sites, we allowed each gene to cast votes for the usage of poly(A) site types according to supporting ESTs. The vote was calculated as follows:



where  is the votes for the usage of poly(A) site type *p *(strong/weak; or 2F/2M/2L; or 3U/3D) in tissue type *t*;  is the number of poly(A/T)-tailed ESTs supporting the usage of poly(A) site type *p *for gene *g *in tissue *t*; and *E*_*g,t *_is the total number of poly(A/T)-tailed ESTs associated with gene *g *in tissue *t*. Percent of usage of a poly(A) type in a tissue (also called observed usage) was calculated by dividing the votes by the total number of genes investigated in the tissue. The median usage for a poly(A) site type is the median of the percent of usage values for the type of all tissues. The distance to the median usage was calculated by (observed usage - median usage)/median usage. A Chi-squared test was performed for each tissue with the null hypothesis that the usage of a given poly(A) site type in the tissue is not different from the median usage. The analyses were carried out in R [49].

### Microarray data analysis of *trans*-acting factors

mRNA expression data were obtained from the NCBI GEO database [47]. The average difference values were normalized to the 75th percentile within each chip. When more than one probe-set mapped to the same gene, the median value was used to represent the mRNA expression level. For tissue types with more than one sample, median values were used. Clustering of tissues and genes with respect to expression profiles of polyadenylation factors were carried out using the Cluster program [50], and presented using TreeView [50].

### Identification of candidate *cis *elements

Genomic regions -100/+100 nt surrounding the poly(A) sites were divided into four sub-regions: -100/-41 nt, -40/-1 nt, +1/+40 nt, and +41/+100 nt. Frequencies of occurrence of all 4,096 hexamers were calculated in each sub-region and in control regions (-300/-200 and +200/+300). Three scores were used to select hexamers in each sub-region: *z*_*un*_, the difference between the frequency of occurrence from poly(A) sites used in the brain and those from poly(A) sites not used in the brain; *z*_*pc*_, the difference between the frequency of occurrence in the sub-region and the frequency of occurrence in the control regions; and *z*_*sw*_, the difference between the frequency of occurrence in the sub-region of strong poly(A) sites and weak poly(A) sites using the 75% cutoff (see above). All z scores were calculated using the following equations. For hexamers in set a and set b, *z*_*ab *_was calculated as follows:



where



and *N*_*a *_and *N*_*b *_are the total number of hexamers associated with set a and set b, respectively. *f*_*a*_*(H) *and *f*_*b*_*(H) *are the frequency of occurrence of hexamer *H *in set a and set b, respectively. A cutoff of 2.5 was used to select hexamers that are significantly biased to one set, which corresponded to a *p* value of approximately 0.01 in a normal distribution. Thus, selection of hexamers by two criteria should result in less than one falsely identified hexamer (4,096 × 0.01 × 0.01 = 0.4). Hexamers that have both *z*_*un *_and *z*_*pc *_above the cutoff, and *z*_*sw *_below the cutoff, were selected for further analysis.

Selected hexamers were grouped based on their mutual distances using the hierarchical clustering function in program R with the average agglomerative method. The distance between two hexamers is their dissimilarity score (*d*) calculated as follows: *d *= 6 - *s*, where *s *is a similarity score. *s *was calculated using a dynamic programming method for global sequence alignment that does not allow gaps, and match and mismatch scores were 1 and 0, respectively. A cutoff of 2.5 was used to group hexamers. Only groups containing more than three hexamers after clustering were selected for further analysis. Hexamers in the same group were aligned using a multiple sequence alignment method using the hexamer with the highest frequency of occurrence as the seed. All other hexamers were aligned to the seed. Aligned hexamers were used to build sequence logos to represent *cis *elements using the Web Logo tool [51]. The height of each nucleotide in a sequence logo reflects the occurrence of the nucleotide in the *cis *element. Aligned hexamers were also used to generate PSSM, which were used to search sequences containing poly(A) sites. For each position in a given *cis *element, the score was calculated by:

*S(n,p) *= log_2_(*f(n,p)*/*f(n)*)

where *S(n,p) *is the score for nucleotide *n *at position *p*, *f(n,p) *is the frequency of occurrence of nucleotide *n *at position *p*, and *f(n) *is the frequency of occurrence of nucleotide *n *in a specific poly(A) region. Sequences with positive scores compared with PSSM were called hits.

## Additional data files

The following additional data are available with the online version of this paper. Additional data file 1 is a PDF containing supplemental tables and figures.

## Supplementary Material

Additional data file 1 A PDF containing supplemental tables and figures. The tables list: Table 1, tissues, cDNA libraries, genes, ESTs, and poly(A) sites used in this study; Table 2, poly(A) site usage of type II genes; Table 3, poly(A) site usage of type III genes; Table 4, usage of strong and weak poly(A) sites using 60% as cutoff; Table 5, usage of strong and weak poly(A) sites using 75% as cutoff; Table 6, usage of strong and weak poly(A) sites using 90% as cutoff. The figures show: Figure 1, Schematic representation of three types of genes; Figure 2, Boxplots of mRNA expression levels of polyadenylation-related factors in brain tissues versus other tissues; Figure 3, selection of hexamers in different poly(A) regions; Figure 4, multiple sequence alignments of PTB and nPTB and CstF-64 and τCstF-64. Click here for file

## Figures and Tables

**Table 1:** Polyadenylation-related protein factors

	Gene name	Gene ID*	U95Av2 probe-sets	U133A probe-sets
1	CPSF-160, cleavage and polyadenylation specificity factor 1, 160 kDa	29894	33132_at	201638_s_at 201639_s_at 33132_at
2	CPSF-100, cleavage and polyadenylation specificity factor 2, 100 kDa	53981	NA	NA
3	CPSF-73, cleavage and polyadenylation specificity factor 3, 73 kDa	51692	NA	NA
4	CPSF-30, cleavage and polyadenylation specificity factor 4, 30 kDa	10898	35743_at	206688_s_at
5	CFI25, cleavage factor Im, 25 kDa	11051	39142_at	202697_at 213461_at
6	CFI68, cleavage factor Im, 68 kDa	11052	35757_at	202469_s_at 202470_s_at
7	hFip1, *Saccharomyces cerevisiae *Fip1p like	81608	NA	221007_s_at
8	CstF50, cleavage stimulatory factor subunit 1, 50 kDa	1477	32723_at	202190_at 32723_at
9	CstF-64, cleavage stimulatory factor subunit 2, 64 kDa	1478	40334_at	204459_at
10	CstF-77, cleavage stimulatory factor subunit 3, 77 kDa	1479	41183_at	203947_at
11	τCstF-64, cleavage stimulatory factor subunit 2, 64 kDa, tau variant	23283	41248_at	212901_s_at 212905_at
12	CFIIA, HEAB, ATP/GTP binding protein, component of CFIIAm [52]	10978	33149_at	204370_at
13	PCF11, pre-mRNA cleavage complex II protein [53]	51585	41665_at	203378_at
14	PABPII, poly(A) binding protein, nuclear 1	8106	39050_at	201544_x_at 201545_s_at 213046_at
15	Symplekin [1,2]	8189	32402_s_at	32402_s_at 202339_at
16	HNRPF, heterogeneous nuclear ribonucleoprotein F [5]	3185	38071_at	201376_s_at
17	HNRPH, heterogeneous nuclear ribonucleoprotein H1 (H) [6]	3187	41292_at	201031_s_at 213470_s_at 213472_at
18	HNRPH', heterogeneous nuclear ribonucleoprotein H2 (H') [6]	3188	41131_f_at 41132_r_at	201132_at
19	U2AF65, U2 small nuclear RNA auxiliary factor2 [7]	11338	32556_at	218381_s_at 218382_s_at
20	U1A, U1 small nuclear ribonucleoprotein polypeptide A [8,10]	6626	40842_at	201770_at
21	PC4, transcriptional coactivator [3]	10923	36171_at	212857_x_at 214512_s_at 221727_at
22	Similar to HSPC182 protein, human HomoloGene of yeast Ssu72 [4]	286528	NA	NA
23	SRp20 [12]	6428	351_f_at 40457_at	202899_s_at 208672_s_at 208673_s_at
24	PTB, polypyrimidine tract binding protein, also known as hnRNP I [11]	5725	40593_at	202189_x_at 211270_x_at 211271_x_at 212015_x_at 212016_s_at 216306_x_at
25	nPTB, polypyrimidine tract binding protein 2	58155	NA	218683_at
26	PAP, poly(A) polymerase	10914	34855_at	209388_at 212718_at 212720_at 215374_at 222035_s_at

References supporting the role of a given factor in mRNA polyadenylation can be found in review articles Proudfoot *et al*. [54], Zhao *et al*. [21], and Edwalds-Gilbert *et al*. [15], if not otherwise noted. Some genes do not have corresponding probe sets on a microarray, as indicated by NA. *Gene IDs are NCBI Entrez Gene IDs [55].
