# Supplementary material for: Biased alternative polyadenylation in human tissues
Source: Genome Biol. 2005 Nov 28;6(12):R100. doi: 10.1186/gb-2005-6-12-r100 (PMC1414089; doi:10.1186/gb-2005-6-12-r100)
Supplement: Additional data file 1 — A PDF containing supplemental tables and figures. The tables list: Table 1, tissues, cDNA libraries, genes, ESTs, and poly(A) sites used in this study; Table 2, poly(A) site usage of type II genes; Table 3, poly(A) site usage of type III genes; Table 4, usage of strong and weak poly(A) sites using 60% as cutoff; Table 5, usage of strong and weak poly(A) sites using 75% as cutoff; Table 6, usage of strong and weak poly(A) sites using 90% as cutoff. The figures show: Figure 1, Schematic representation of three types of genes; Figure 2, Boxplots of mRNA expression levels of polyadenylation-related factors in brain tissues versus other tissues; Figure 3, selection of hexamers in different poly(A) regions; Figure 4, multiple sequence alignments of PTB and nPTB and CstF-64 and τCstF-64. [file gb-2005-6-12-r100-S1.pdf]

ADDITIONAL DATA FILE 1

Supplemental Table 1. Tissues, cDNA libraries, genes, ESTs, and poly(A) sites used in this study.
Supplemental Table 2. Poly(A) site usage of type II genes.
Supplemental Table 3. Poly(A) site usage of type III genes.
Supplemental Table 4. Usage of strong and weak poly(A) sites using 60% as cutoff.
Supplemental Table 5. Usage of strong and weak poly(A) sites using 75% as cutoff.
Supplemental Table 6. Usage of strong and weak poly(A) sites using 90% as cutoff.

Supplemental Figure 1. Schematic representation of three types of genes.
Supplemental Figure 2. Boxplots of mRNA expression levels of polyadenylation-related factors in brain tissues versus other tissues. (A) U95Av2 data. (B) U133A data. For each plot, values from brain tissues are on the left, and those from other tissues are on the right.
Supplemental Figure 3. Selection of hexamers in different poly(A) regions. (A) –100/-41, (B) –40/-1, (C) +1/+40, and (D) +41/+100. For each panel, a two-dimensional scatter-plot for z_{un} and z_{pc} is shown on the left, and clustering of hexamers is shown on the right. In each scatter-plot, x-axis is z_{un}, and y-axis is z_{pc}. Each dot represents a hexamer. Cutoffs of 2.5 were used for both z_{un} and z_{pc}, as indicated by vertical and horizontal lines, respectively. Hexamers in green are the ones with z_{sw} >2.5. Hexamers in red are the selected ones for clustering. Sequence logos of *cis* elements are shown under corresponding hexamer groups.
Supplemental Figure 4. Multiple sequence alignments of PTB and nPTB (A), and CstfF-64 and τCstF-64 (B). Only the isoform a of PTB (NP_002810) was used here. ClustalW was used to align protein sequences with default settings. Identical residues in the alignment are marked with asterisks "*"; strongly and weakly conserved residues are marked with colons ":" and periods ".", respectively. The protein sequence accessions for nPTB, CstfF-64 and τCstF-64 are NP_067013, NP_001316, and NP_056050, respectively.

Supplemental Table 1. Tissues, cDNA libraries, genes, ESTs, and poly(A) sites used in this study.

Tissue	# of lib	# of genes	# of ESTs	# of PA
adipose	3	115	137	115
adrenal cortex	2	269	321	273
blood	28	747	1903	791
bone	8	3944	11057	4454
bone marrow	10	1337	3535	1410
brain	48	2226	5717	2355
cerebrum	13	251	321	253
cervix	5	811	1372	833
colon	50	1795	3039	1881
ear	1	1213	2576	1283
endocrine	5	569	875	580
esophagus	1	302	356	304
eye	14	3626	8896	3989
genitourinary	6	382	446	390
germ cell	4	415	522	421
head and neck	34	726	1205	740
heart	7	46	64	47
kidney	14	1533	2692	1605
liver	13	1380	3617	1460
lung	31	2859	8012	3068
lymph/lymph node	8	1299	2425	1343
mammary gland	37	507	1721	513
muscle	7	1302	2474	1354
nervous	9	276	315	282
ovary	25	2035	5596	2133
pancreas	13	2541	5566	2718
pancreatic islet	6	2509	9769	2759
peripheral nervous system	6	984	1276	1007
pineal gland	2	154	185	155
pituitary gland	5	157	259	158
placenta	28	3690	8539	4092
prostate	32	2044	5505	2183
retina	10	3117	5697	3367
skin	17	2280	6753	2436
soft tissue	5	537	681	545
spleen	3	263	415	267
stomach	34	2569	5284	2736
testis	11	1168	1854	1218
thymus	1	18	18	18
thyroid	30	387	558	394
uterus	16	4253	10844	4775
vascular	7	356	548	363

# of lib: number of non-normalized libraries
# of genes: number of genes studied
# of ESTs: number of poly(A/T)-tailed ESTs from non-normalized libraries
# of PA: number of poly(A) sites

Supplemental Table 2. Poly(A) site usage of type II genes.

Tissue	# of genes	# of ESTs	Vf	Vm	Vl	Pf	Pm	Pl	Df	Dm	Dl	Pval
adipose	48	59	19	9	20	0.40	0.19	0.42	0.19	-0.07	-0.11	6.31E-01
adrenal cortex	108	123	29.5	25	53.5	0.27	0.23	0.50	-0.18	0.15	0.06	4.16E-01
blood	267	797	120.39	52.46	94.15	0.45	0.20	0.35	0.36	-0.03	-0.25	6.99E-05
bone	1476	4404	464.85	296.12	715.03	0.31	0.20	0.48	-0.05	-0.01	0.03	3.62E-01
bone marrow	554	1407	156	136.53	261.47	0.28	0.25	0.47	-0.15	0.22	0.01	7.77E-03
brain	794	1969	239.44	147.82	406.74	0.30	0.19	0.51	-0.09	-0.08	0.09	4.26E-02
cerebrum	96	116	21.5	19.5	55	0.22	0.20	0.57	-0.33	0.01	0.22	6.16E-02
cervix	317	498	109.47	73.58	133.95	0.35	0.23	0.42	0.04	0.15	-0.10	2.14E-01
colon	689	1122	233.52	120.2	335.28	0.34	0.17	0.49	0.02	-0.14	0.04	2.12E-01
ear	534	1272	129.33	134.22	270.45	0.24	0.25	0.51	-0.27	0.25	0.08	2.70E-05
endocrine	234	337	79.27	42.23	112.5	0.34	0.18	0.48	0.02	-0.11	0.02	7.31E-01
esophagus	91	110	28	24	39	0.31	0.26	0.43	-0.07	0.31	-0.09	3.31E-01
eye	1285	3207	444.93	264.81	575.26	0.35	0.21	0.45	0.04	0.02	-0.05	3.37E-01
genitourinary	159	188	50.83	41	67.17	0.32	0.26	0.42	-0.04	0.28	-0.10	1.93E-01
germ cell	144	188	55.45	24.75	63.8	0.39	0.17	0.44	0.16	-0.15	-0.06	3.55E-01
head and neck	276	451	94.75	52.33	128.92	0.34	0.19	0.47	0.03	-0.06	0.00	8.55E-01
heart	13	14	6	0	7	0.46	0.00	0.54	0.39	-1.00	0.15	1.82E-01
kidney	581	1003	181.81	112.83	286.36	0.31	0.19	0.49	-0.06	-0.04	0.05	4.73E-01
liver	510	1287	179.63	108.86	221.51	0.35	0.21	0.43	0.06	0.06	-0.07	3.18E-01
lung	995	2705	349.04	186.12	459.84	0.35	0.19	0.46	0.06	-0.07	-0.02	3.26E-01
lymph node	505	958	167.09	116.3	221.61	0.33	0.23	0.44	0.00	0.14	-0.07	2.20E-01
mammary gland	188	549	59.59	36	92.41	0.32	0.19	0.49	-0.05	-0.05	0.05	8.08E-01
muscle	484	877	155.1	98.34	230.57	0.32	0.20	0.48	-0.04	0.01	0.01	8.81E-01
nervous	116	137	23.67	30.33	62	0.20	0.26	0.53	-0.39	0.30	0.14	1.22E-02
ovary	754	1987	290.11	160.77	303.12	0.38	0.21	0.40	0.16	0.06	-0.14	8.83E-04
pancreas	950	2226	293.09	199.88	457.03	0.31	0.21	0.48	-0.07	0.04	0.02	3.31E-01
pancreatic islet	1003	3765	275.13	216.25	511.61	0.27	0.22	0.51	-0.17	0.07	0.09	6.87E-04
peripheral nervous system	353	444	112.33	67	173.67	0.32	0.19	0.49	-0.04	-0.06	0.05	6.56E-01
pineal gland	58	74	20.5	11.5	26	0.35	0.20	0.45	0.06	-0.02	-0.04	9.33E-01
pituitary gland	57	82	20	14	23	0.35	0.25	0.40	0.06	0.22	-0.14	5.69E-01
placenta	1428	3066	511.66	315.95	600.39	0.36	0.22	0.42	0.08	0.10	-0.10	1.59E-03
prostate	808	2069	267.26	167.68	373.06	0.33	0.21	0.46	0.00	0.03	-0.02	8.90E-01
retina	1106	2021	419.34	213.41	473.26	0.38	0.19	0.43	0.14	-0.04	-0.09	2.66E-03
skin	852	2396	286.6	166.91	398.49	0.34	0.20	0.47	0.01	-0.03	0.00	9.04E-01
soft tissue	206	258	55.6	38.4	112	0.27	0.19	0.54	-0.19	-0.08	0.16	7.93E-02
spleen	82	118	27.8	13.2	41	0.34	0.16	0.50	0.02	-0.20	0.07	6.52E-01
stomach	999	2137	325.36	195.85	477.78	0.33	0.20	0.48	-0.02	-0.03	0.02	7.95E-01
testis	436	686	160.61	94.33	181.06	0.37	0.22	0.42	0.11	0.07	-0.12	8.64E-02
thymus	6	6	2	1	3	0.33	0.17	0.50	0.00	-0.17	0.07	9.76E-01
thyroid	162	214	52	34	76	0.32	0.21	0.47	-0.03	0.04	0.00	9.46E-01
uterus	1607	4300	487.28	365.07	754.65	0.30	0.23	0.47	-0.09	0.13	0.00	1.04E-02
vascular	156	221	61.2	30.5	64.3	0.39	0.20	0.41	0.18	-0.03	-0.12	2.44E-01

# of genes: number of genes studied
# of ESTs: number of poly(A/T)-tailed ESTs from non-normalized libraries
Vf, Vm, and Vl: votes for 2F, 2M, and 2L poly(A) sites, respectively
Pf, Pm, and Pl: percent of usage of 2F, 2M, and 2L poly(A) sites, respectively
Df, Dm, and Dl: distance to the median usage for 2F, 2M, and 2L poly(A) sites, respectively
Pval: p-value from Chi-squared test

Supplemental Table 3. Poly(A) site usage of type III genes.

Tissue	# of genes	# of ESTs	Vu	Vd	Pu	Pd	Du	Dd	Pval
adipose	23	28	3	20	0.13	0.87	0.11	-0.02	8.41E-01
adrenal cortex	64	85	3	61	0.05	0.95	-0.60	0.08	8.09E-02
blood	172	380	37.22	134.78	0.22	0.78	0.85	-0.11	5.02E-05
bone	1085	3219	140.59	944.41	0.13	0.87	0.11	-0.01	1.98E-01
bone marrow	373	984	39.63	333.37	0.11	0.89	-0.09	0.01	5.17E-01
brain	516	1274	72.51	443.49	0.14	0.86	0.20	-0.03	9.67E-02
cerebrum	64	79	2	62	0.03	0.97	-0.73	0.10	3.28E-02
cervix	187	333	19	168	0.10	0.90	-0.13	0.02	5.12E-01
colon	423	761	48.67	374.33	0.12	0.88	-0.02	0.00	9.00E-01
ear	397	790	37.2	359.8	0.09	0.91	-0.20	0.03	1.48E-01
endocrine	121	201	9.06	111.94	0.07	0.93	-0.36	0.05	1.49E-01
esophagus	73	78	14	59	0.19	0.81	0.64	-0.08	4.69E-02
eye	955	2271	193.63	761.37	0.20	0.80	0.73	-0.10	1.69E-16
genitourinary	91	108	5	86	0.05	0.95	-0.53	0.07	6.55E-02
germ cell	108	140	8.5	99.5	0.08	0.92	-0.33	0.04	2.15E-01
head and neck	163	243	13.83	149.17	0.08	0.92	-0.27	0.04	2.01E-01
heart	15	27	2.5	12.5	0.17	0.83	0.42	-0.06	5.50E-01
kidney	378	658	46.93	331.07	0.12	0.88	0.06	-0.01	6.66E-01
liver	318	980	40.93	277.07	0.13	0.87	0.10	-0.01	5.17E-01
lung	676	1770	97.92	578.08	0.14	0.86	0.24	-0.03	2.44E-02
lymph node	329	587	37.65	291.35	0.11	0.89	-0.02	0.00	8.84E-01
mammary gland	101	214	12	89	0.12	0.88	0.02	0.00	9.55E-01
muscle	331	576	38.14	292.86	0.12	0.88	-0.02	0.00	9.19E-01
nervous	67	76	3.5	63.5	0.05	0.95	-0.55	0.07	9.90E-02
ovary	479	1079	66.35	412.65	0.14	0.86	0.18	-0.02	1.43E-01
pancreas	621	1207	56.05	564.95	0.09	0.91	-0.23	0.03	3.80E-02
pancreatic islet	615	1878	78.24	536.76	0.13	0.87	0.09	-0.01	4.31E-01
peripheral nervous system	256	360	25	231	0.10	0.90	-0.17	0.02	3.35E-01
pineal gland	29	38	1	28	0.03	0.97	-0.71	0.09	1.67E-01
pituitary gland	33	44	2	31	0.06	0.94	-0.48	0.06	3.13E-01
placenta	1008	2164	192.25	815.75	0.19	0.81	0.63	-0.08	3.34E-13
prostate	478	1269	70.76	407.24	0.15	0.85	0.27	-0.04	3.49E-02
retina	777	1391	167.6	609.4	0.22	0.78	0.84	-0.11	1.15E-17
skin	540	1485	99	441	0.18	0.82	0.57	-0.08	1.63E-06
soft tissue	152	202	9	143	0.06	0.94	-0.49	0.07	2.66E-02
spleen	57	82	8	49	0.14	0.86	0.20	-0.03	5.84E-01
stomach	645	1182	77.35	567.65	0.12	0.88	0.02	0.00	8.19E-01
testis	327	530	47.56	279.44	0.15	0.85	0.24	-0.03	1.10E-01
thymus	5	5	0	5	0.00	1.00	-1.00	0.13	4.16E-01
thyroid	93	138	6.05	86.95	0.07	0.93	-0.44	0.06	1.19E-01
uterus	1123	2833	137.08	985.92	0.12	0.88	0.04	-0.01	5.99E-01
vascular	79	132	6.5	72.5	0.08	0.92	-0.30	0.04	3.37E-01

# of genes: number of genes studied
# of ESTs: number of poly(A/T)-tailed ESTs from non-normalized libraries
Vu and Vd: votes for 3U and 3D poly(A) sites, respectively
Pu and Pd: percent of usage of 3U and 3D poly(A) sites, respectively
Du and Dd, distance to the median usage for 3U and 3D poly(A) sites, respectively
Pval: p-value from Chi-squared test

Supplemental Table 4. Usage of strong and weak poly(A) sites using 60% as cutoff

Tissue	# of genes	# of ESTs	Vs	Vw	Ps	Pw	Ds	Dw	Pval
adipose	38	48	34.00	4.00	0.89	0.11	0.07	-0.36	3.23E-01
adrenal cortex	90	110	78.00	12.00	0.87	0.13	0.04	-0.19	4.22E-01
blood	226	589	185.03	40.97	0.82	0.18	-0.02	0.10	5.02E-01
bone	1019	4195	829.78	189.22	0.81	0.19	-0.03	0.13	7.08E-02
bone marrow	461	1516	397.09	63.91	0.86	0.14	0.03	-0.16	1.31E-01
brain	582	1733	479.79	102.21	0.82	0.18	-0.01	0.07	4.78E-01
cerebrum	93	112	77.00	16.00	0.83	0.17	-0.01	0.04	8.49E-01
cervix	251	487	214.15	36.85	0.85	0.15	0.02	-0.11	4.45E-01
colon	522	1005	433.76	88.24	0.83	0.17	-0.01	0.03	7.89E-01
ear	433	1130	363.49	69.51	0.84	0.16	0.00	-0.03	8.15E-01
endocrine	199	299	171.17	27.83	0.86	0.14	0.03	-0.15	3.45E-01
esophagus	77	96	61.17	15.83	0.79	0.21	-0.05	0.25	3.33E-01
eye	898	2756	671.49	226.51	0.75	0.25	-0.10	0.53	1.52E-12
genitourinary	112	146	98.97	13.03	0.88	0.12	0.06	-0.29	1.68E-01
germ cell	138	201	121.30	16.70	0.88	0.12	0.05	-0.27	1.66E-01
head and neck	227	417	192.75	34.25	0.85	0.15	0.02	-0.08	5.75E-01
heart	16	18	13.00	3.00	0.81	0.19	-0.03	0.14	8.06E-01
kidney	444	888	368.80	75.20	0.83	0.17	-0.01	0.03	7.91E-01
liver	407	1387	336.78	70.22	0.83	0.17	-0.01	0.05	6.70E-01
lung	742	2561	619.08	122.92	0.83	0.17	0.00	0.01	9.44E-01
lymph node	384	863	313.17	70.83	0.82	0.18	-0.02	0.12	2.97E-01
mammary gland	167	343	143.83	23.17	0.86	0.14	0.03	-0.16	3.66E-01
muscle	349	760	290.98	58.02	0.83	0.17	0.00	0.01	9.38E-01
nervous	87	110	75.17	11.83	0.86	0.14	0.03	-0.17	4.70E-01
ovary	535	1476	445.82	89.18	0.83	0.17	0.00	0.01	9.01E-01
pancreas	707	1955	603.82	103.18	0.85	0.15	0.02	-0.11	1.79E-01
pancreatic islet	694	3437	571.21	122.79	0.82	0.18	-0.01	0.07	3.85E-01
peripheral nervous system	286	403	239.17	46.83	0.84	0.16	0.00	-0.01	9.65E-01
pineal gland	46	64	41.00	5.00	0.89	0.11	0.07	-0.34	3.06E-01
pituitary gland	56	76	45.67	10.33	0.82	0.18	-0.02	0.12	6.90E-01
placenta	913	2224	688.29	224.71	0.75	0.25	-0.10	0.49	3.29E-11
prostate	601	2015	506.66	94.34	0.84	0.16	0.01	-0.05	6.09E-01
retina	700	1426	478.71	221.29	0.68	0.32	-0.18	0.92	3.38E-27
skin	645	2354	526.08	118.92	0.82	0.18	-0.02	0.12	1.78E-01
soft tissue	172	234	147.42	24.58	0.86	0.14	0.03	-0.13	4.41E-01
spleen	81	126	71.13	9.87	0.88	0.12	0.05	-0.26	2.98E-01
stomach	706	1798	592.65	113.35	0.84	0.16	0.00	-0.03	7.66E-01
testis	339	658	272.58	66.42	0.80	0.20	-0.04	0.19	1.21E-01
thymus	6	6	6.00	0.00	1.00	0.00	0.20	-1.00	2.77E-01
thyroid	132	186	111.25	20.75	0.84	0.16	0.01	-0.05	8.16E-01
uterus	1062	3835	848.95	213.05	0.80	0.20	-0.04	0.22	1.60E-03
vascular	144	230	128.52	15.48	0.89	0.11	0.07	-0.35	6.42E-02

# of genes: number of genes studied
# of ESTs: number of poly(A/T)-tailed ESTs from non-normalized libraries
Vs and Vw: votes for strong and weak poly(A) sites, respectively
Ps and Pw: percent of usage of strong and weak poly(A) sites, respectively
Ds and Dw, distance to the median usage for strong and weak poly(A) sites, respectively
Pval: p-value from Chi-squared test

Supplemental Table 5. Usage of strong and weak poly(A) sites using 75% as cutoff

Tissue	# of genes	# of ESTs	Vs	Vw	Ps	Pw	Ds	Dw	Pval
adipose	32	42	31.00	1.00	0.97	0.03	0.08	-0.69	1.93E-01
adrenal cortex	67	84	61.00	6.00	0.91	0.09	0.01	-0.11	7.66E-01
blood	170	509	151.20	18.80	0.89	0.11	-0.01	0.10	6.60E-01
bone	657	3338	579.88	77.12	0.88	0.12	-0.02	0.17	1.49E-01
bone marrow	325	1262	298.36	26.64	0.92	0.08	0.02	-0.18	2.67E-01
brain	422	1469	370.88	51.12	0.88	0.12	-0.02	0.21	1.58E-01
cerebrum	69	87	64.00	5.00	0.93	0.07	0.03	-0.28	4.39E-01
cervix	188	404	165.65	22.35	0.88	0.12	-0.02	0.18	4.01E-01
colon	362	786	316.76	45.24	0.88	0.12	-0.03	0.24	1.21E-01
ear	284	856	259.99	24.01	0.92	0.08	0.02	-0.16	3.72E-01
endocrine	154	244	138.67	15.33	0.90	0.10	0.00	-0.01	9.70E-01
esophagus	55	70	49.50	5.50	0.90	0.10	0.00	0.00	9.91E-01
eye	575	2052	448.77	126.23	0.78	0.22	-0.13	1.19	2.14E-21
genitourinary	86	116	77.30	8.70	0.90	0.10	0.00	0.01	9.83E-01
germ cell	113	175	104.80	8.20	0.93	0.07	0.03	-0.28	3.24E-01
head and neck	175	361	159.75	15.25	0.91	0.09	0.01	-0.13	5.58E-01
heart	13	14	12.00	1.00	0.92	0.08	0.03	-0.23	7.78E-01
kidney	314	717	277.56	36.44	0.88	0.12	-0.02	0.16	3.58E-01
liver	280	1151	247.49	32.51	0.88	0.12	-0.02	0.16	3.84E-01
lung	500	2121	450.38	49.62	0.90	0.10	0.00	-0.01	9.28E-01
lymph node	269	723	232.34	36.66	0.86	0.14	-0.04	0.36	5.06E-02
mammary gland	132	299	118.83	13.17	0.90	0.10	0.00	-0.01	9.79E-01
muscle	243	626	216.38	26.62	0.89	0.11	-0.01	0.09	6.37E-01
nervous	61	82	57.17	3.83	0.94	0.06	0.04	-0.38	3.28E-01
ovary	370	1215	332.66	37.34	0.90	0.10	0.00	0.00	9.77E-01
pancreas	469	1528	431.07	37.93	0.92	0.08	0.02	-0.19	1.58E-01
pancreatic islet	468	2897	413.89	54.11	0.88	0.12	-0.02	0.15	2.75E-01
peripheral nervous system	199	299	182.50	16.50	0.92	0.08	0.02	-0.17	4.10E-01
pineal gland	31	47	29.00	2.00	0.94	0.06	0.04	-0.36	5.06E-01
pituitary gland	46	64	38.00	8.00	0.83	0.17	-0.08	0.73	9.75E-02
placenta	559	1545	460.70	98.30	0.82	0.18	-0.08	0.75	3.04E-09
prostate	420	1705	370.82	49.18	0.88	0.12	-0.02	0.17	2.57E-01
retina	425	877	308.34	116.66	0.73	0.27	-0.19	1.73	7.77E-33
skin	448	2011	394.63	53.37	0.88	0.12	-0.02	0.19	1.89E-01
soft tissue	123	169	112.67	10.33	0.92	0.08	0.02	-0.16	5.43E-01
spleen	69	111	62.47	6.53	0.91	0.09	0.01	-0.06	8.72E-01
stomach	464	1403	415.78	48.22	0.90	0.10	0.00	0.03	8.04E-01
testis	230	513	201.29	28.71	0.88	0.12	-0.03	0.24	2.19E-01
thymus	5	5	5.00	0.00	1.00	0.00	0.11	-1.00	4.55E-01
thyroid	96	139	87.75	8.25	0.91	0.09	0.02	-0.14	6.36E-01
uterus	673	3002	582.28	90.72	0.87	0.13	-0.04	0.34	3.04E-03
vascular	110	192	102.52	7.48	0.93	0.07	0.04	-0.32	2.57E-01

# of genes: number of genes studied
# of ESTs: number of poly(A/T)-tailed ESTs from non-normalized libraries
Vs and Vw: votes for strong and weak poly(A) sites, respectively
Ps and Pw: percent of usage of strong and weak poly(A) sites, respectively
Ds and Dw, distance to the median usage for strong and weak poly(A) sites, respectively
Pval: p-value from Chi-squared test

Supplemental Table 6. Usage of strong and weak poly(A) sites using 90% as cutoff

Tissue	# of genes	# of ESTs	Vs	Vw	Ps	Pw	Ds	Dw	Pval
adipose	19	27	19.00	0.00	1.00	0.00	0.04	-1.00	3.60E-01
adrenal cortex	36	49	34.50	1.50	0.96	0.04	0.00	-0.01	9.88E-01
blood	91	379	86.31	4.69	0.95	0.05	-0.01	0.22	6.56E-01
bone	225	2143	215.40	9.60	0.96	0.04	0.00	0.01	9.70E-01
bone marrow	135	914	130.98	4.02	0.97	0.03	0.01	-0.29	4.74E-01
brain	167	966	160.25	6.75	0.96	0.04	0.00	-0.04	9.11E-01
cerebrum	28	40	28.00	0.00	1.00	0.00	0.04	-1.00	2.67E-01
cervix	92	266	89.05	2.95	0.97	0.03	0.01	-0.24	6.30E-01
colon	142	445	136.88	5.12	0.96	0.04	0.01	-0.14	7.17E-01
ear	112	527	110.39	1.61	0.99	0.01	0.03	-0.66	1.43E-01
endocrine	88	148	84.00	4.00	0.95	0.05	0.00	0.08	8.78E-01
esophagus	28	39	25.00	3.00	0.89	0.11	-0.07	1.54	8.71E-02
eye	196	1118	176.39	19.61	0.90	0.10	-0.06	1.37	5.52E-05
genitourinary	43	63	40.50	2.50	0.94	0.06	-0.02	0.38	6.02E-01
germ cell	60	112	58.55	1.45	0.98	0.02	0.02	-0.43	4.88E-01
head and neck	103	261	99.42	3.58	0.97	0.03	0.01	-0.18	7.08E-01
heart	8	9	8.00	0.00	1.00	0.00	0.04	-1.00	5.53E-01
kidney	152	458	143.97	8.03	0.95	0.05	-0.01	0.25	5.13E-01
liver	139	815	131.03	7.97	0.94	0.06	-0.02	0.36	3.73E-01
lung	205	1435	194.64	10.36	0.95	0.05	-0.01	0.20	5.51E-01
lymph node	111	478	103.76	7.24	0.93	0.07	-0.02	0.55	2.27E-01
mammary gland	74	225	70.33	3.67	0.95	0.05	-0.01	0.18	7.51E-01
muscle	104	412	99.89	4.11	0.96	0.04	0.00	-0.06	8.93E-01
nervous	27	43	25.67	1.33	0.95	0.05	-0.01	0.17	8.54E-01
ovary	163	822	154.20	8.80	0.95	0.05	-0.01	0.28	4.53E-01
pancreas	185	1021	179.88	5.12	0.97	0.03	0.02	-0.34	3.27E-01
pancreatic islet	175	1835	165.67	9.33	0.95	0.05	-0.01	0.26	4.63E-01
peripheral nervous system	90	155	88.50	1.50	0.98	0.02	0.03	-0.60	2.29E-01
pineal gland	16	26	16.00	0.00	1.00	0.00	0.04	-1.00	4.01E-01
pituitary gland	26	40	24.00	2.00	0.92	0.08	-0.04	0.82	3.78E-01
placenta	197	725	182.42	14.58	0.93	0.07	-0.03	0.76	2.62E-02
prostate	176	1124	165.63	10.37	0.94	0.06	-0.02	0.40	2.69E-01
retina	128	322	111.19	16.81	0.87	0.13	-0.09	2.11	5.18E-07
skin	185	1411	177.40	7.60	0.96	0.04	0.00	-0.03	9.41E-01
soft tissue	69	105	65.67	3.33	0.95	0.05	-0.01	0.14	8.01E-01
spleen	40	75	38.80	1.20	0.97	0.03	0.01	-0.29	7.02E-01
stomach	174	824	167.85	6.15	0.96	0.04	0.01	-0.16	6.54E-01
testis	94	296	84.30	9.70	0.90	0.10	-0.06	1.45	3.24E-03
thymus	3	3	3.00	0.00	1.00	0.00	0.04	-1.00	7.16E-01
thyroid	57	94	55.50	1.50	0.97	0.03	0.02	-0.38	5.52E-01
uterus	227	1724	213.62	13.38	0.94	0.06	-0.02	0.40	2.09E-01
vascular	69	132	68.00	1.00	0.99	0.01	0.03	-0.66	2.53E-01

# of genes: number of genes studied
# of ESTs: number of poly(A/T)-tailed ESTs from non-normalized libraries
Vs and Vw: votes for strong and weak poly(A) sites, respectively
Ps and Pw: percent of usage of strong and weak poly(A) sites, respectively
Ds and Dw, distance to the median usage for strong and weak poly(A) sites, respectively
Pval: p-value from Chi-squared test

Supplemental Figure 1.

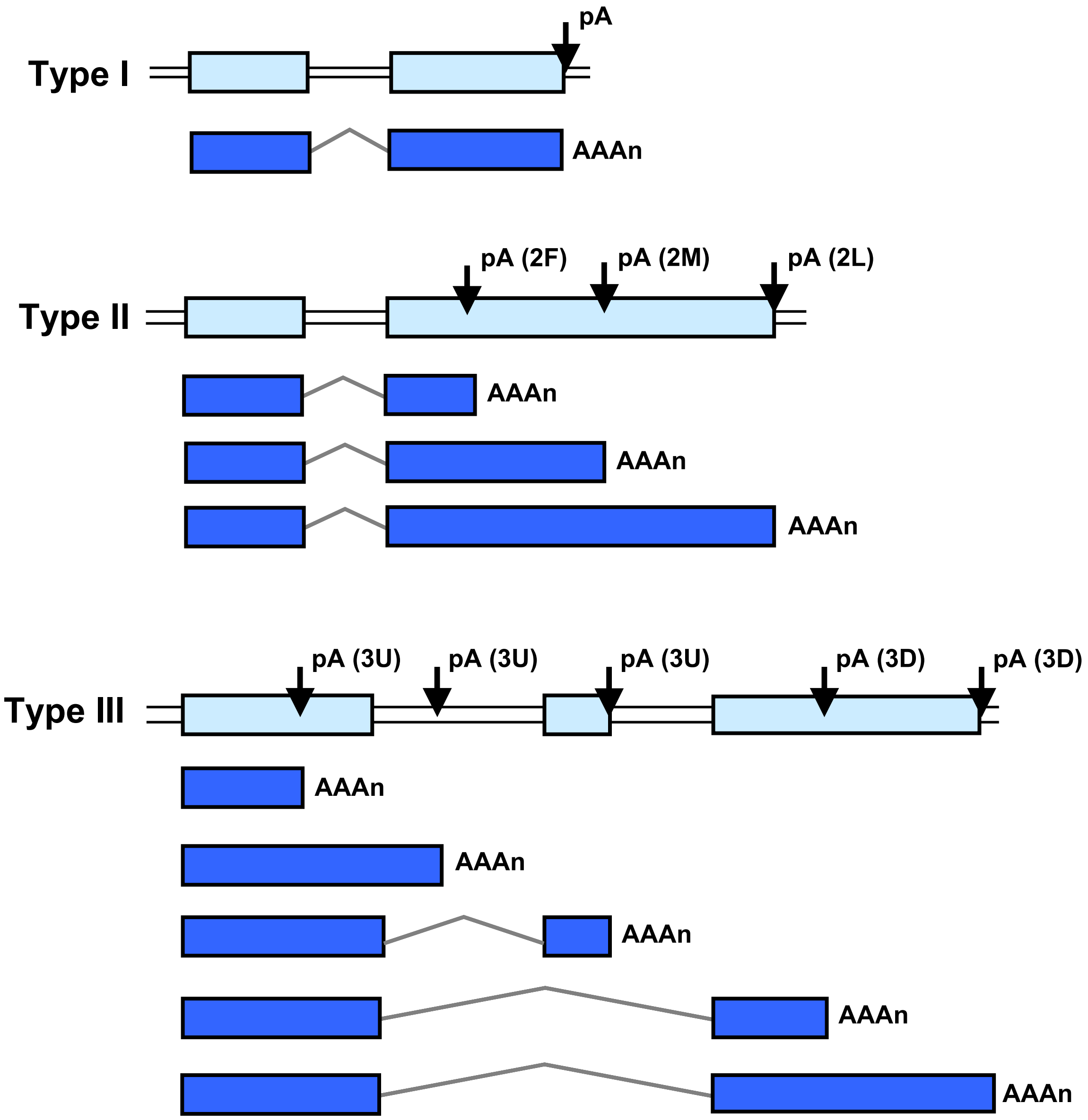

Supplemental Figure 2.

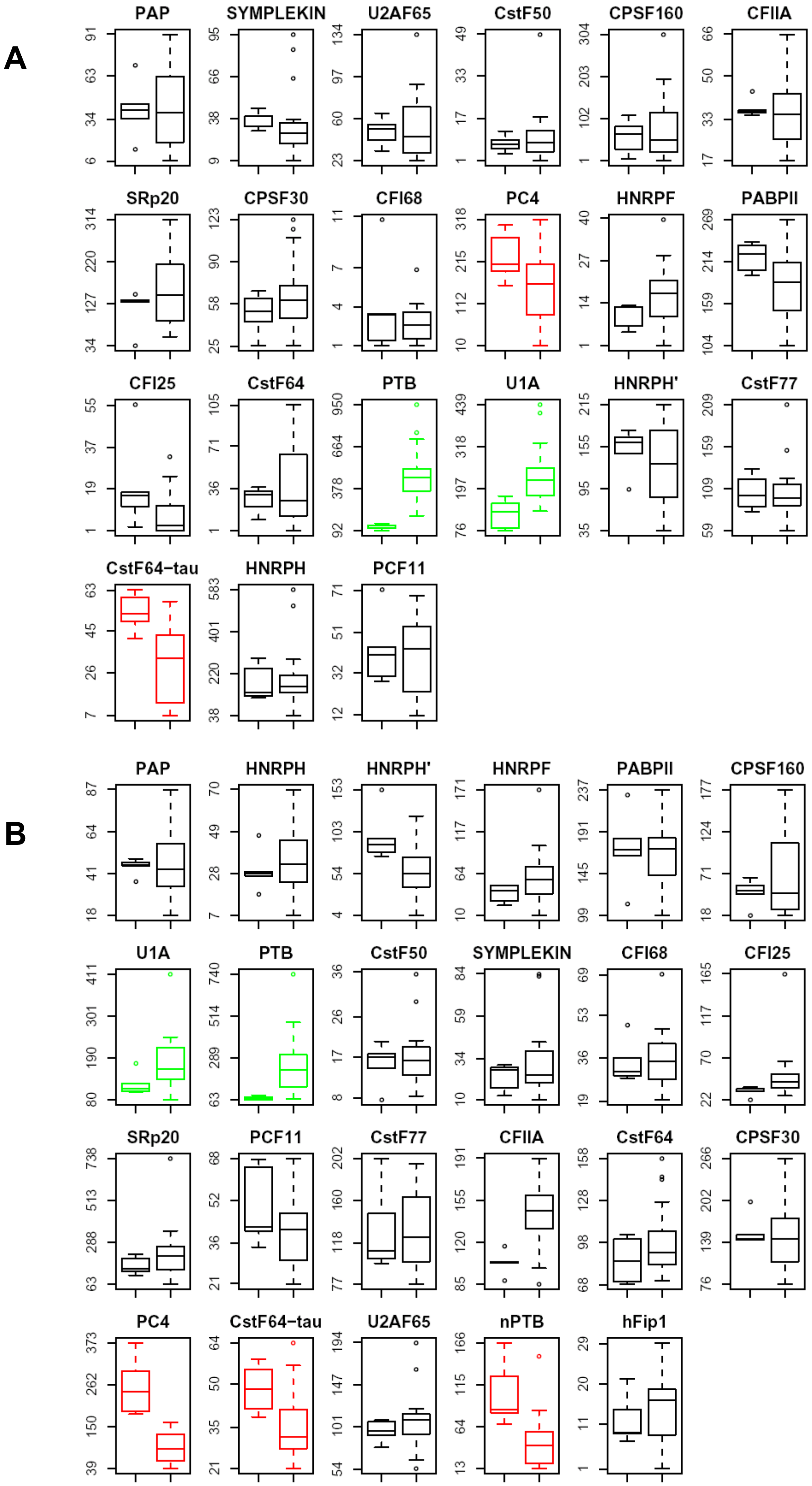

Supplemental Figure 3.

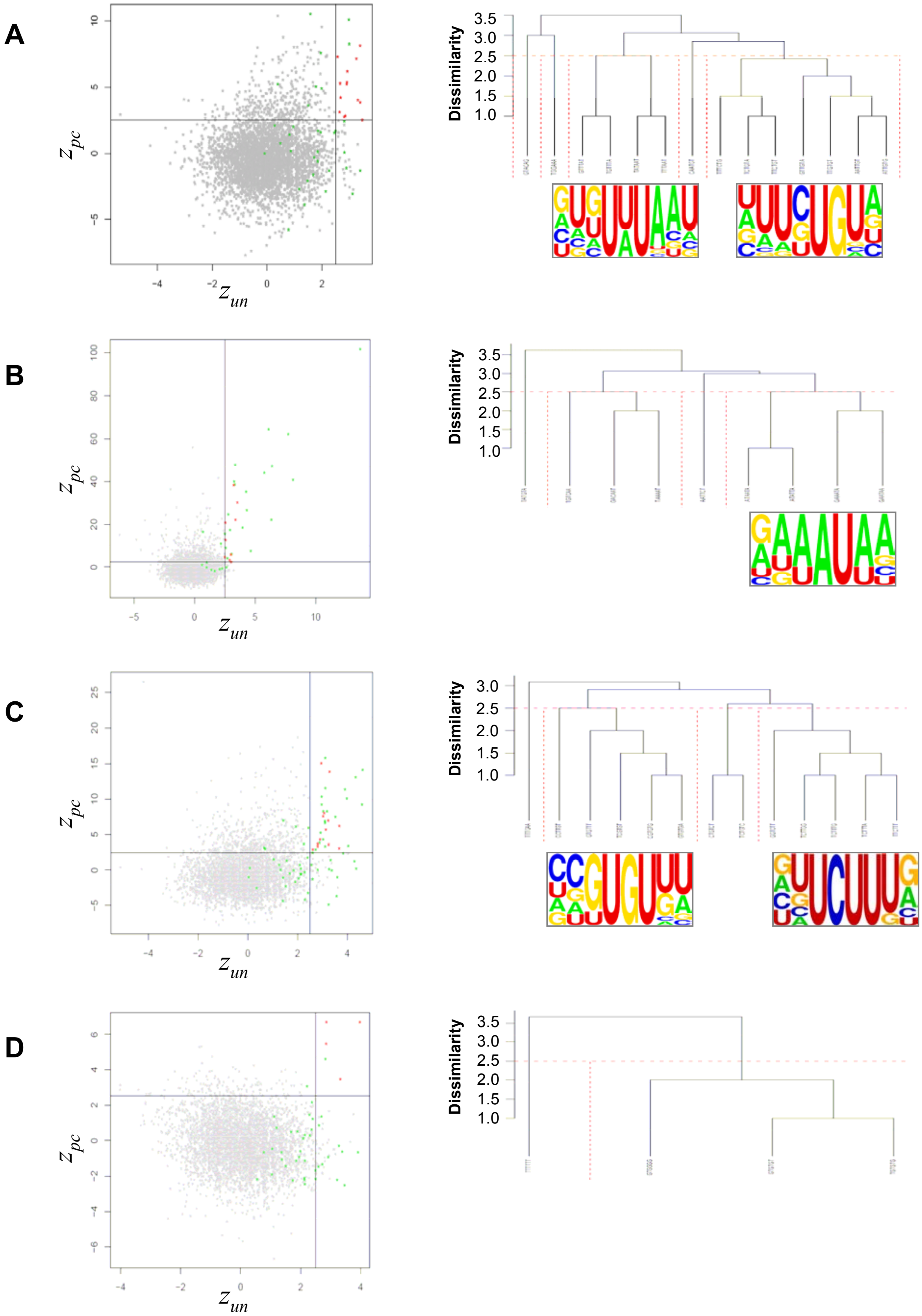

Supplemental Figure 4.

A

```
     *****.::***.*******:* .* ..*    *. . :***********:.:  *.****:****** :*** ***:***
 PTB MDGIVPDIAVGTKRGSDELFSTCVTNGPFIMSSNSASAANGNDSKKFKGDSRSAGVPSRVIHIRKLPIDVTEGEVISLGL   80
nPTB MDGIVTEVAVGVKRGSDELLSGSVLSSPNSNMSSMVVTANGNDSKKFKGEDKMDGAPSRVLHIRKLPGEVTETEVIALGL   80

     *******:**********:*: ***** ******::*** **.******:*********.: *  ****.****.:**:.
 PTB PFGKVTNLLMLKGKNQAFIEMNTEEAANTMVNYYTSVTPVLRGQPIYIQFSNHKELKTDSSPNQARAQAALQAVNSVQSG  160
nPTB PFGKVTNILMLKGKNQAFLELATEEAAITMVNYYSAVTPHLRNQPIYIQYSNHKELKTDNTLN-QRAQAVLQAVTAVQTA  159

     * .*:.  ::*. . . ..********::*::****************:*******************.***.**:***:
 PTB NLALAASAAAVDAGMAMAGQSPVLRIIVENLFYPVTLDVLHQIFSKFGTVLKIITFTKNNQFQALLQYADPVSAQHAKLS  240
nPTB NTPLSG--TTVSESAVTPAQSPVLRIIIDNMYYPVTLDVLHQIFSKFGAVLKIITFTKNNQFQALLQYGDPVNAQQAKLA  237

     ********************..**********************.**:** ::****.
 PTB LDGQNIYNACCTLRIDFSKLTSLNVKYNNDKSRDYTRPDLPSGDSQPSLDQTMAAAFGAPGIISASPYAGAGFPPTFAIP  320
nPTB LDGQNIYNACCTLRIDFSKLVNLNVKYNNDKSRDYTRPDLPSGDGQPALDPAIAAAFAK--------------------  296

     ::: *:**   ***:*****  :**********:.:**::..**:******** * ******* **************:*
 PTB QAAGLSVPNVHGALAPLAIP--SAAAAAAAAGRIAIPGLAGAGNSVLLVSNLNPERVTPQSLFILFGVYGDVQRVKILFN  398
nPTB ETSLLAVP---GALSPLAIPNAAAAAAAAAAGRVGMPGVSAGGNTVLLVSNLNEEMVTPQSLFTLFGVYGDVQRVKILYN  373

     **:.**:*******:****.****:*::** **:******.******* :*******:**********************
 PTB KKENALVQMADGNQAQLAMSHLNGHKLHGKPIRITLSKHQNVQLPREGQEDQGLTKDYGNSPLHRFKKPGSKNFQNIFPP  478
nPTB KKDSALIQMADGNQSQLAMNHLNGQKMYGKIIRVTLSKHQTVQLPREGLDDQGLTKDFGNSPLHRFKKPGSKNFQNIFPP  453

     *************:****:.**:..**.**.***** *:****:**.:****:********::****************
 PTB SATLHLSNIPPSVSEEDLKVLFSSNGGVVKGFKFFQKDRKMALIQMGSVEEAVQALIDLHNHDLGENHHLRVSFSKSTI  557
nPTB SATLHLSNIPPSVAEEDLRTLFANTGGTVKAFKFFQ-DHKMALLQMATVEEAIQALIDLHNYNLGENHHLRVSFSKSTI  531
```

B

```
        *:.*:*****:*****************************.***************************************
 CstF64 MAGLTVRDPAVDRSLRSVFVGNIPYEATEEQLKDIFSEVGPVVSFRLVYDRETGKPKGYGFCEYQDQETALSAMRNLNGR   80
tCstF64 MSSLAVRDPAMDRSLRSVFVGNIPYEATEEQLKDIFSEVGSVVSFRLVYDRETGKPKGYGFCEYQDQETALSAMRNLNGR   80

        *************************..**:*:****:.*.********::************************ *****
 CstF64 EFSGRALRVDNAASEKNKEELKSLGTGAPVIESPYGETISPEDAPESISKAVASLPPEQMFELMKQMKLCVQNSPQEARN  160
tCstF64 EFSGRALRVDNAASEKNKEELKSLGPAAPIIDSPYGDPIDPEDAPESITRAVASLPPEQMFELMKQMKLCVQNSHQEARN  160

        *********************:***********: ::..**.*:.*.*  :***.*.        ** :***** ***.*
 CstF64 MLLQNPQLAYALLQAQVVMRIVDPEIALKILHRQTNIPTLIAGNPQPVHGAGPGSGS--------NVSMNQQNPQAPQAQ  232
tCstF64 MLLQNPQLAYALLQAQVVMRIMDPEIALKILHRKIHVTPLIPGKSQSVSVSGPGPGPGPGLCPGPNVLLNQQNPPAPQPQ  240

         *.   *:. *****:.:***:**** :****.*.*****:***.**.*:**** ***.:***** *.****.: **.:.
 CstF64 SLGGMHVNGAPPLMQASMQGGVPAPGQMPAAVTGPGPGSLAPGGGMQAQVGMPGSGPVSMERGQVPMQDPRAAMQRGSLP  312
tCstF64 HLARRPVKDIPPLMQTPIQGGIPAPGPIPAAVPGAGPGSLTPGGAMQPQLGMPGVGPVPLERGQVQMSDPRAPIPRGPVT  320

        ..  .*****************************************..**::***..**:***** :** *:.******:
 CstF64 ANVPTPRGLLGDAPNDPRGGTLLSVTGEVEPRGYLGPPHQGPPMHHVPGHESRGPPPHELRGGPLPEPRPLMAEPRGPML  392
tCstF64 PGGLPPRGLLGDAPNDPRGGTLLSVTGEVEPRGYLGPPHQGPPMHHASGHDTRGPSSHEMRGGPLGDPRLLIGEPRGPMI  400

        **** *:*******     :*.**:****:          ..:*:*.** *.**: ***:*.**.***:***:: *****
 CstF64 DQRGPPLDGRGGRDPRGIDARGMEARAMEARGLDARGLEARAMEARAMEARAMEARAMEARAMEVRGMEARGMDTRGPVP  472
tCstF64 DQRGLPMDGRGGRD-----SRAMETRAMET----------EVLETRVMERRGMETCAMETRGMEARGMDARGLEMRGPVP  465

        ..***:..*:***.***:**  * **.****                :*********.****
 CstF64 GPRGPIPSGMQGPSPINMGAVVP-QGSRQVP---------------VMQGTGMQGASIQGG-------------------  517
tCstF64 SSRGPMTGGIQGPGPINIGAGGPPQGPRQVPGISGVGNPGAGMQGTGIQGTGMQGAGIQGGGMQGAGIQGVSIQGGGIQG  545

                   ***..*****.******:*****************************************.
 CstF64 -----------SQPGGFSPGQNQVTPQDHEKAALIMQVLQLTADQIAMLPPEQRQSILILKEQIQKSTGAP  577
tCstF64 GGIQGASKQGGSQPSSFSPGQSQVTPQDQEKAALIMQVLQLTADQIAMLPPEQRQSILILKEQIQKSTGAS  616
```
